# Immediate excitatory effect of transcranial ultrasound intensity on the human primary motor cortex: a double-blind, sham-controlled and crossover study

**DOI:** 10.3389/fnhum.2026.1884060

**Published:** 2026-08-21

**Authors:** Shu Cheng, Moxian Chen, Shan Liang, Yanpin Zhao, Xiaojing Long, De Meng, Feifei Zhu, Jing Zhang, Zhongfei Bai, Lijuan Ao

**Affiliations:** 1 School of Rehabilitation, Kunming Medical University, Kunming, China; 2 Department of Rehabilitation Medicine, China Resources and Wisco General Hospital, Wuhan University of Science and Technology, Wuhan, China; 3 Department of Neurology and Neurological Rehabilitation, Shanghai Yangzhi Rehabilitation Hospital (Shanghai Sunshine Rehabilitation Center), School of Medicine, Tongji University, Shanghai, China; 4 Department of Pediatric Rehabilitation Center, Shanghai Yangzhi Rehabilitation Hospital (Shanghai Sunshine Rehabilitation Center), School of Medicine, Tongji University, Shanghai, China; 5 Shenzhen Institute of Advance Technology, Chinese Academy of Sciences, Shenzhen, China

**Keywords:** focused ultrasound, motor cortex, neuroplasticity, spatial peak pulse average intensity, transcranial magnetic stimulation

## Abstract

**Objective:**

To systematically characterize the intensity-dependent effects of low-intensity transcranial ultrasound stimulation (TUS) on excitability in the human primary motor cortex (M1) and to identify an effective intensity threshold for inducing immediate excitatory changes.

**Methods:**

In a randomized, double-blind, sham-controlled crossover design, twenty-five healthy right-handed adults (mean age, 24.0 ± 3.3 years) received 20 min of 500 kHz TUS to their M1 hotspot. Five sessions were conducted, each employing a different spatial peak pulse average intensity (ISPPA: 3, 6, 9, or 12 W/cm^2^ or sham stimulation in the free-field). Corticospinal and intracortical excitability were assessed immediately before and after sonication using single and paired-pulse transcranial magnetic stimulation (TMS) protocols to measure motor-evoked potential (MEP) amplitudes, short and long-interval intracortical inhibition (SICI/LICI), intracortical facilitation (ICF), and cortical silent period (cSP).

**Results:**

A clear intensity-dependent effect was observed. Only sonication at 12 W/cm^2^ significantly increased MEP amplitudes (*p* = 0.036), indicating enhanced corticospinal excitability. TUS also significantly shortened the cSP duration at intensities of 6 (*p* = 0.039), 9 (*p* = 0.036), and 12 (*p* = 0.003) W/cm^2^compared with pre-TUS. Notably, these changes occurred without any significant modulation of intracortical measures (SICI, LICI, and ICF) across all conditions. The observed excitatory effect was not correlated with baseline MEP amplitude or gender.

**Conclusion:**

Our findings demonstrate that TUS modulates human M1 excitability in a selective and intensity-dependent manner, identifying an ISPPA of 12 W/cm^2^ as a critical threshold for driving corticospinal potentiation. This provides a rational basis for parameter selection in future TUS protocols and advances its development as a precision tool for therapeutic applications in neurological disorders.

**Clinical trial registration:**

Identifier, ChiCTR2600125507.

## Introduction

Non-invasive brain stimulation (NIBS) approaches, including transcranial magnetic stimulation (TMS) and transcranial direct current stimulation (tDCS), have become cornerstone tools for investigating brain function and developing novel therapies for neurological and psychiatric disorders (Somaa et al., 2022; Xia et al., 2025; Dastgheib et al., 2025). A well-established dichotomy exists within these modalities, where low-frequency TMS (Chen et al., 1997; Baur et al., 2020; Nardone et al., 2020) or cathodal tDCS (Frey and Malaty, 2025; Greenwell et al., 2025) generally elicit lasting inhibition of cortical excitability, whereas high-frequency TMS (Wang et al., 2025; van Hattem et al., 2025) or anodal tDCS(Grange et al., 2025; Xue et al., 2025; Jiang et al., 2025) produce sustained increases in cortical excitability. Despite their widespread use, these established techniques are constrained by limited spatial resolution and an inability to non-invasively access deep brain structures (Romanella et al., 2020). Emerging as a powerful complementary solution, low-intensity transcranial ultrasound stimulation (TUS) overcomes these limitations by offering high-precision, focal neuromodulation with the unique capability to target subcortical regions (Xia et al., 2025). This has catalyzed a surge of research exploring its therapeutic potential across a spectrum of conditions, including depression (Mesik et al., 2024; Guinjoan, 2023; Fan et al., 2024), Parkinson’s disease (PD)(Zhong et al., 2023; Gasca-Salas et al., 2021), Alzheimer’s disease (Rezai et al., 2024; Rezai et al., 2020), neuropathic pain (Zhang et al., 2025), epilepsy (Bubrick et al., 2022), and disorder of consciousness (DOC) (Cain et al., 2022), with studies consistently affirming its safety and capacity to modulate neuronal activity and network connectivity (Feng and Li, 2024; Toccaceli et al., 2019; Mahoney et al., 2023; Kaloss et al., 2022).

However, the clinical translation of TUS is critically dependent on establishing a clear and predictable relationship between sonication parameters and neurophysiological outcomes. Unlike the established frequency-dependent rules of TMS, the parameter space of TUS remains poorly understood, leading to highly inconsistent findings in the literature. For instance, while one study by Zadeh et al. (2024) reported that pulse repetition frequencies (PRFs) of 10 and 100 Hz significantly suppressed motor cortex excitability, others found no effect of PRF, instead identifying specific duty cycles (10%) and sonication duration (0.4–0.5 s) as the key parameters for inducing inhibition (Fomenko et al., 2020). Compounding this complexity, research by Zeng et al. demonstrated that 5 Hz-repetitive bursts were optimal for enhancing cortical excitability, contrasting with the inhibitory effects often reported (Zeng et al., 2024). Amidst this uncertainty, one of the most critical yet underexplored parameters is the Spatial Peak Pulse Average Intensity (ISPPA). As a primary determinant of the acoustic energy delivered to neural tissue, ISPPA is not only a critical index of safety but is also fundamentally linked to functional efficacy (Qin et al., 2024; Pasquinelli et al., 2019), a principle supported by preclinical studies in animal models, such as rat and sheep, showing its significant impact on ion channel function and neuronal firing (Lee et al., 2016; Tipsawat et al., 2022; Kim et al., 2014). Nevertheless, there is a striking paucity of research systematically investigating how varying ISPPA levels affect human motor cortical excitability.

To address this critical knowledge gap, the present study was designed to characterize the immediate effects of TUS intensity on the human primary motor cortex (M1). We investigated how four distinct ISPPA levels (3, 6, 9, and 12 W/cm^2^ in the free-field) and a sham condition modulate corticospinal and intracortical excitability. Using a neuronavigation-guided, single and paird-pulse TMS-electromyogram (EMG) paradigm, we performed a sequential assessment of cortical silent period (cSP), short-interval intracortical inhibition (SICI), motor-evoked potential (MEP) amplitude, intracortical facilitation (ICF), and long-interval intracortical inhibition (LICI). By elucidating the intensity dependent relationship between acoustic intensity and cortical excitability, this study aims to define TUS parameters with reliable excitatory effects, thereby providing a foundational step toward optimizing TUS protocols for future therapeutic and basic science applications.

## Materials and methods

### Participants

A total of twenty-five healthy, right-handed subjects (19 females, 6 males; mean age ± standard deviation = 24.0 ± 3.3 years, range = 21–30 years) recruited from Shanghai Yangzhi Rehabilitation Hospital participated in this study. Right-handedness was confirmed using the Edinburgh Handedness Inventory (Veale, 2014). Exclusion criteria encompassed any history of neurological or psychiatric disorders, current use of neuropsychotropic medications, sleep deprivation, and any contraindications to TMS or TUS (Rossi et al., 2011), as assessed by a safety screening questionnaire. The study protocol was approved by the local Institutional Review Board and conformed to the principles of the Declaration of Helsinki.

### TUS waveform and quantitative acoustic field mapping

Transcranial ultrasonic waveforms were produced by a single-element ultrasonic transducer (GreenValley BrainTech Medical Technology Co. Ltd.) with a 48 mm aperture diameter and a radius of curvature of 43 mm. To precisely characterize the acoustic field, a quantitative mapping procedure was performed in a degassed, deionized water tank. The acoustic pressure profile was measured with a calibrated needle hydrophone (NH1000, Precision Acoustics Corporation), connected to a preamplifier (PA18081, Precision Acoustics Corporation). The hydrophone was mounted on a computer-controlled three-axis positioning stage, allowing for precise spatial sampling. The transducer, fitted with a water-filled acoustic collimator sealed a sound-transmitting membrane, positioned opposite the hydrophone.

Custom LabVIEW software (National Instruments) controlled the three-axis stage for automated scanning and data acquisition. An initial axial scan (z-axis) was conducted to determine the focal distance. Subsequently, a high-resolution planar scan (12.3 × 12.3 mm) was performed at this focal depth to map the x-y acoustic pressure distribution. Finally, a cross-sectional scan (20.5 × 50.5 mm) in the x-z plane was acquired to visualize the pressure profile along the beam axis, ensuring accurate characterization of the focal volume and the peak pressure location.

### TMS-EMG

**Figure 1:**
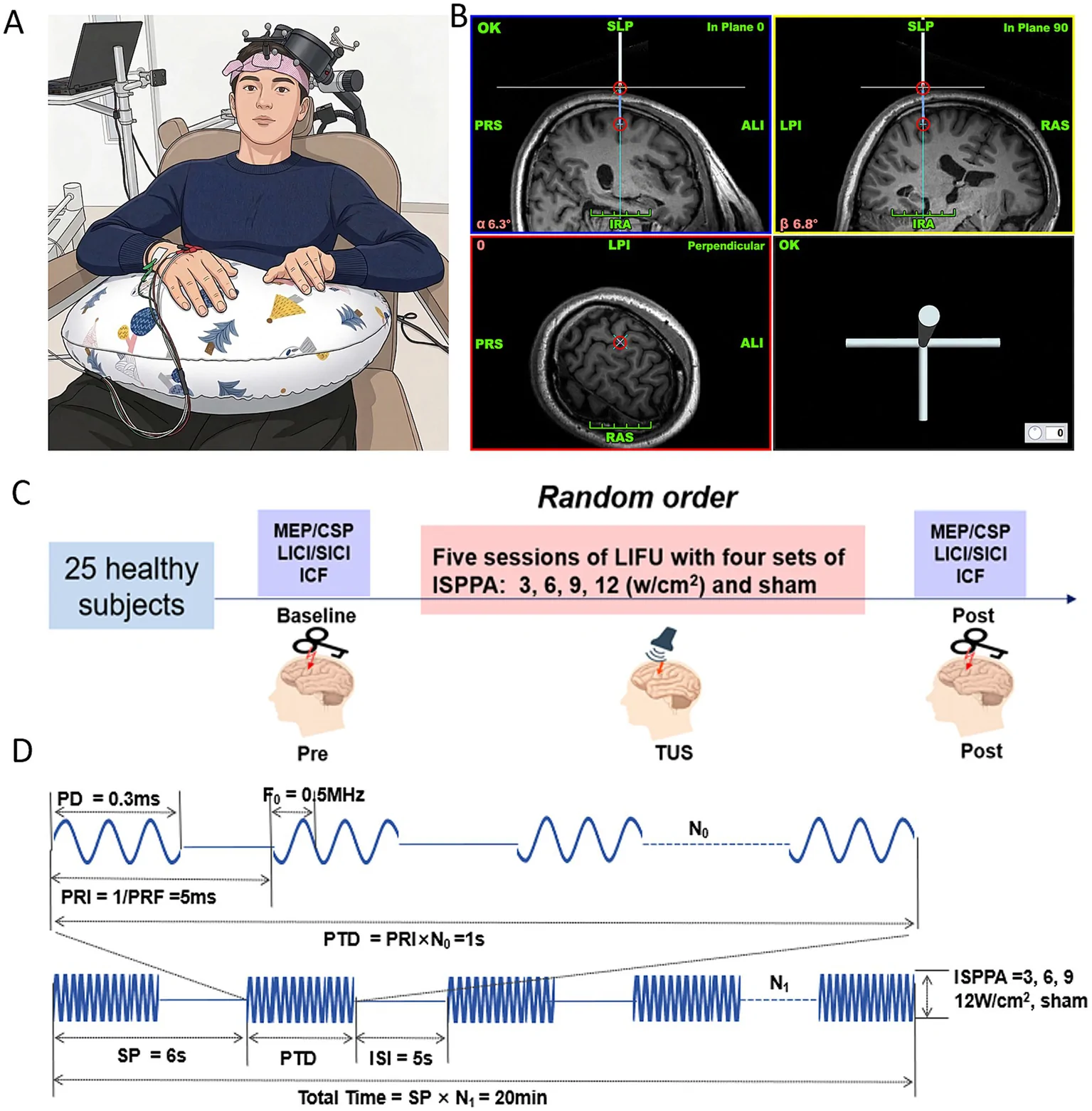
**(A)** Schematic of TMS coil orientation over the left motor cortex. The coil was positioned at ~45° relative to the midline with the handle directed posteriorly and laterally. **(B)** Neuronavigation setup for precise targeting. The position of the coil over the scalp was continuously monitored, with the red sphere indicating the M1 target based on a standardized MRI template. **(C)** Experimental design. Participants underwent five sessions (four active TUS intensities and one sham condition) in a randomized order, separated by a minimum one week washout period. Cortical excitability measures (cSP, SICI, MEP, ICF, LICI) were assessed sequentially at baseline and immediately after sonication (post-TUS). **(D)** TUS stimulation parameters. Key parameters included: Pulse Duration (*PD*) = 0.3 ms, Pulse Repetition Interval (*PRI*) = 5 ms, Pulse Train Duration (*PTD*) = 1 s, Inter-Stimulus Interval (*ISI*) = 5 s, resulting in Sonication Period (*SP*) = 6 s, *PRF* = 200 Hz, *DC* = 6%. Sonication was delivered for a total of 20 min. The four active intensity levels (*ISPPA* = 3, 6, 9, 12 W/cm^2^) and a sham condition were examined.

All TMS procedures were performed over the M1 of the dominant (left) hemispheres. Biphasic TMS pulses were delivered using a figure-of-eight cooling coil (Cooling B-65, wing diameter 75 mm) connected to a magnetic stimulator (model X100 option, MagVenture A/S, Denmark). To elicit optimal motor responses, the coil was positioned tangentially to the scalp with its handle pointing posteriorly and laterally at an approximate 45° angle to the mid-sagittal line (Di Lazzaro et al., 2008) (Figure 1A). A stereotactic neuronavigation system (Northern Digital Inc., Canada), co-registered to a standard brain template, was used to continuously monitor and maintain precise coil positioning throughout the experiment (Figure 1B). The motor hotspot was functionally defined as the scalp location that consistently produced the largest MEPs in the contralateral first dorsal interosseous (FDI) muscle. Surface EMG signals were recorded from the right FDI muscle using disposable Ag-AgCl electrodes arranged in a belly–tendon montage. A ground electrode was placed over the ulnar styloid process of the wrist. The raw EMG signal was amplified, filtered, and digitized for offline analysis.

### Experimental protocol

This study employed a randomized, double-blind, sham-controlled, crossover design (Figure 1C). Each participant completed five experimental sessions, corresponding to four active ISPPA levels (3, 6, 9, and 12 W/cm^2^ in the free-field) and one sham condition. The order of sessions was randomized for each participant, and both participants and the data analyst were blinded to the stimulation condition. A minimum washout period of 1 week was enforced between consecutive sessions, with the entire experiment completed within 2 months. The sham condition was used, where the active face of the transducer was directed toward the target, but minimum dosage acoustic energy (ISPPA = 0.08 W/cm^2^ in the free-field) was delivered. In each session, TUS was applied for a total duration of 20 minutes to the M1 hotspot previously identified by TMS. To ensure stable targeting, participants were instructed to remain still once the transducer was positioned. A conductive gel (Jinya® Multi-Purpose Ultrasound Gel, Tianjin, China) was used to ensure optimal acoustic coupling between the ultrasound transducer and the scalp. The transducer was carefully oriented to ensure the acoustic beam was as perpendicular as possible to the local skull surface, thereby minimizing beam refraction and aberration.

An electronic control system drove the transducer to generate ultrasonic pulses characterized by an operating frequency of 500 kHz, a pulse duration of 300 μs, a pulse repetition interval of 5 ms, and a pulse train duration of 1 s, with each pulse train delivered every 6 s. The spatial-peak temporal-average intensities (ISPTA) for the four active conditions were 180, 360, 540, and 720 mW/cm^2^, respectively, in addition to the sham stimulation (Figure 1D). These values are accordance with the International Transcranial Ultrasonic Stimulation Safety and Standards consortium (ITRUSST) safety consensus (Aubry et al., 2025). Sonication was delivered for a total of 20 min. During sonication and the pre and post assessment periods, participants were instructed to remain awake, relaxed, and fixate on a plus sign placed directly in front of them, and their wakefulness was continuously observed by the experimenter. The TUS-induced changes in corticospinal and intracortical excitability were assessed with TMS-EMG immediately before (baseline) and after (post-TUS) sonication in each session. Gaussian white noise played through earphones served to mask the transducer’s auditory stimulus. To monitor safety, participants were instructed to report any discomfort at any time during the experiment.

### TMS-EMG recording

A series of TMS-EMG measurements were conducted at baseline and post TUS. Post TUS single and paired pulse TMS measurements were initiated within 1 min after completion of the TUS protocol. The resting motor threshold (RMT) was determined at the beginning of each session and defined as the lowest stimulator intensity (expressed as a percentage of maximal stimulator output, or % MSO) that produced peak-to-peak MEP amplitudes exceeding 50 μV in at least five out of 10 consecutive trials. To measure corticospinal excitability, single pulse MEPs were elicited using a test stimulus (TS) intensity set at 120% of the individual’s RMT. SICI and ICF were examined by delivering a subthreshold conditioning stimulus (80% of RMT) at interpulse intervals of 2 ms and 10 ms, respectively, before the TS. LICI was evaluated using a pair pulse paradigm of two consecutive TS pulses separated by 100 ms. The cSP was measured as the duration of EMG silence following a suprathreshold TS delivered while the participant maintained a voluntary contraction of the thumb-index finger (FDI) muscle at 30% of their maximum (Werhahn et al., 1999). For each of these protocols, 20 trials were performed with a 5-s inter-trial interval.

### Data processing and analysis

The TMS-EMG signals were processed offline using custom scripts in MATLAB (The MathWorks, Inc., Natick, MA) (Bai et al., 2023a, 2023b). Raw EMG signals, digitized at 5 kHz, were bandpass filtered using a fourth-order Butterworth filter (10-2000 Hz). The signals were then epoched into trials (from −1,000 ms to 999 ms) and baseline corrected using the pre-stimulus interval (−500 ms to −20 ms). Peak to peak MEP amplitudes were measured for each trial. The duration of cSP was measured from the onset of the TMS pulse to the first point within a 5 ms window where 50% of EMG signal samples returned to a level at least three times the standard deviation above the mean EMG of the silent period (Bai et al., 2023a, 2023b). For the paired pulse protocols, ICF, SICI, and LICI were expressed as a ratio to the MEP amplitude elicited by the TS. Finally, the data from all trials for each condition were averaged to derive the grand mean value for subsequent statistical analysis.

### Statistical analysis

All statistics analyses were performed using SPSS software (version 25, SPSS Inc., United States), with the alpha level set at 0.05 (two-tailed). Baseline TMS-EMG measurements across the five sessions were compared using one way analysis of variance (ANOVA) for normal distributed data, or a Kruskal-Wallis (KW) rank sum test for non-normally distributed data. The primary effects of TUS were analyzed using a two-way repeated measures ANOVA (rmANOVA) within subject factors of Time (pre-TUS and post-TUS) and Condition (sham, 3, 6, 9, 12 W/cm^2^). The the main effects (Time and Condition) and interaction effect (Time × Condition) were also interest. Mauchly’s test was used to evaluate the sphericity assumption; if violated, the Greenhouse–Geisser correction was applied. Significant interactions were followed up with Bonferroni corrected *post hoc* comparisons. Spearman and point biserial correlation analyses were used to assess the relationship between the TUS induced change in MEP amplitude (△MEP) at the 12 W/cm^2^ intensity and baseline MEP amplitude and gender, respectively.

## Results

### Acoustic beam properties of focused ultrasound

**Figure 2:**
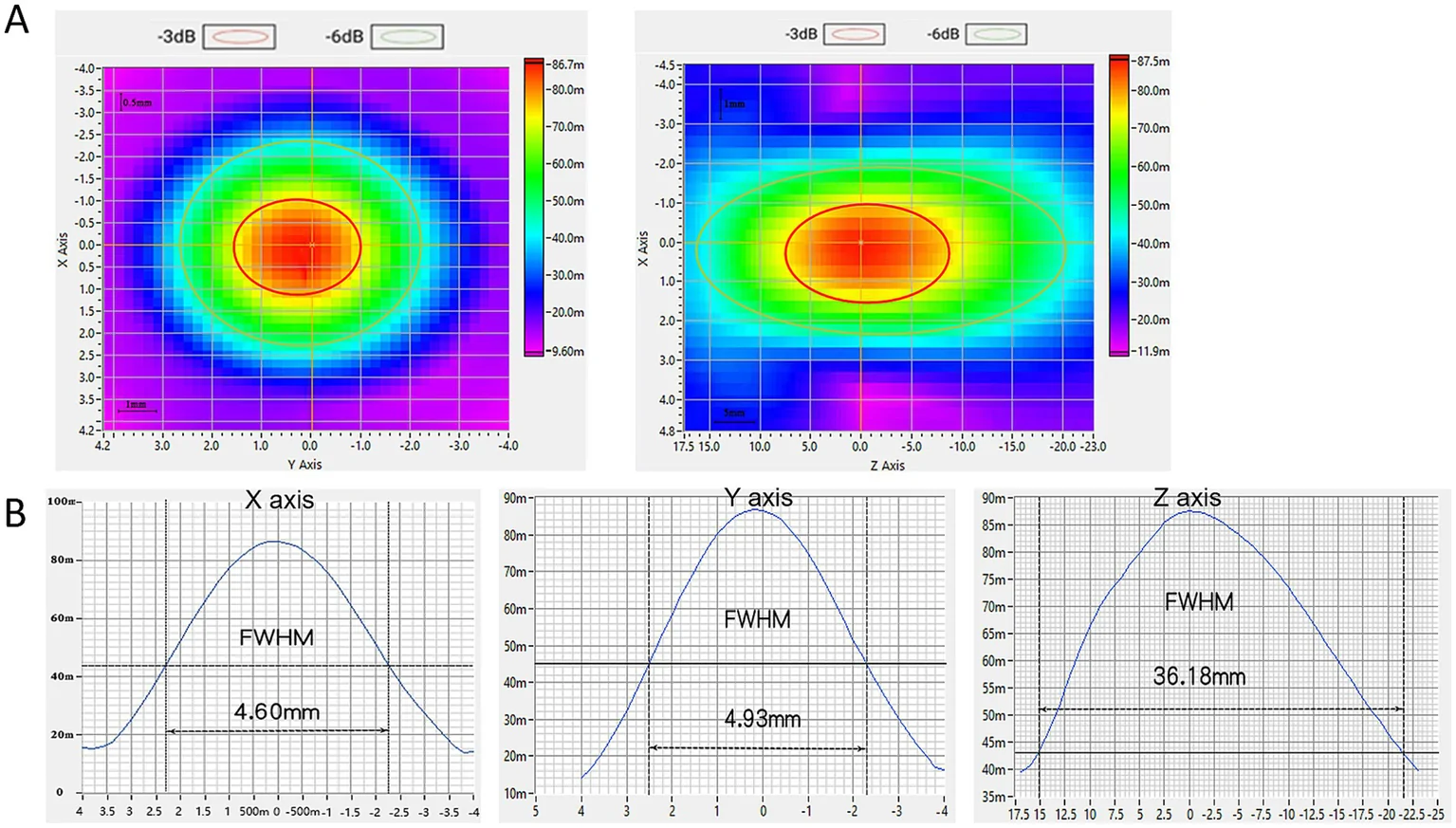
Acoustic pressure field characterization of the 0.5 MHz transducer. **(A)** Acoustic intensity profiles in the transverse (*x*-*y* plane) and sagittal (*x*-*z* plane) dimensions, measured in free water. The color map represents normalized acoustic pressure. **(B)** Line plots showing the normalized acoustic pressure along the lateral (*x*, left), vertical (*y*, middle), and axial (*z*, right) through the focal point. The FWHM of the focal spot was 4.60 mm (lateral), 4.93 mm (vertical), and 36.18 mm along the propagation path.

The acoustic pressure field generated by the TUS transducer was quantified in degassed, deionized water using a calibrated hydrophone mounted on a motorized 3-axis positioning system. Figure 2A illustrates the acoustic beam profile in the transverse plane (XY) and the sagittal plane (ZX) relative to the sonication path. The full width at half maximum (FWHM) of acoustic pressure at the focus was 4.60 mm in the lateral dimension (*x* axis), 4.93 mm in the vertical dimension (*y* axis), and 36.18 mm in the axial dimension (*z* axis) (Figure 2B). This focused spatial profile ensured precise targeting of the motor cortex. The ISPPA used in this study ranged from 3 to 12 W/cm^2^, which are well below the US Food and Drug Administration (FDA) safety guideline of 190 W/cm^2^. The corresponding ISPTA ranged from 180 to 720 mW/cm^2^, which are within the FDA recommendation limit of 720 mW/cm^2^ (Martin et al., 2024). During the experiment, one participant reported mild, transient distending pain over the right frontal region during the 9 W/cm^2^ sonication, which resolved spontaneously after 3 min rest period. No other adverse events were reported.

### TMS-EMG measures

**Figure 3:**
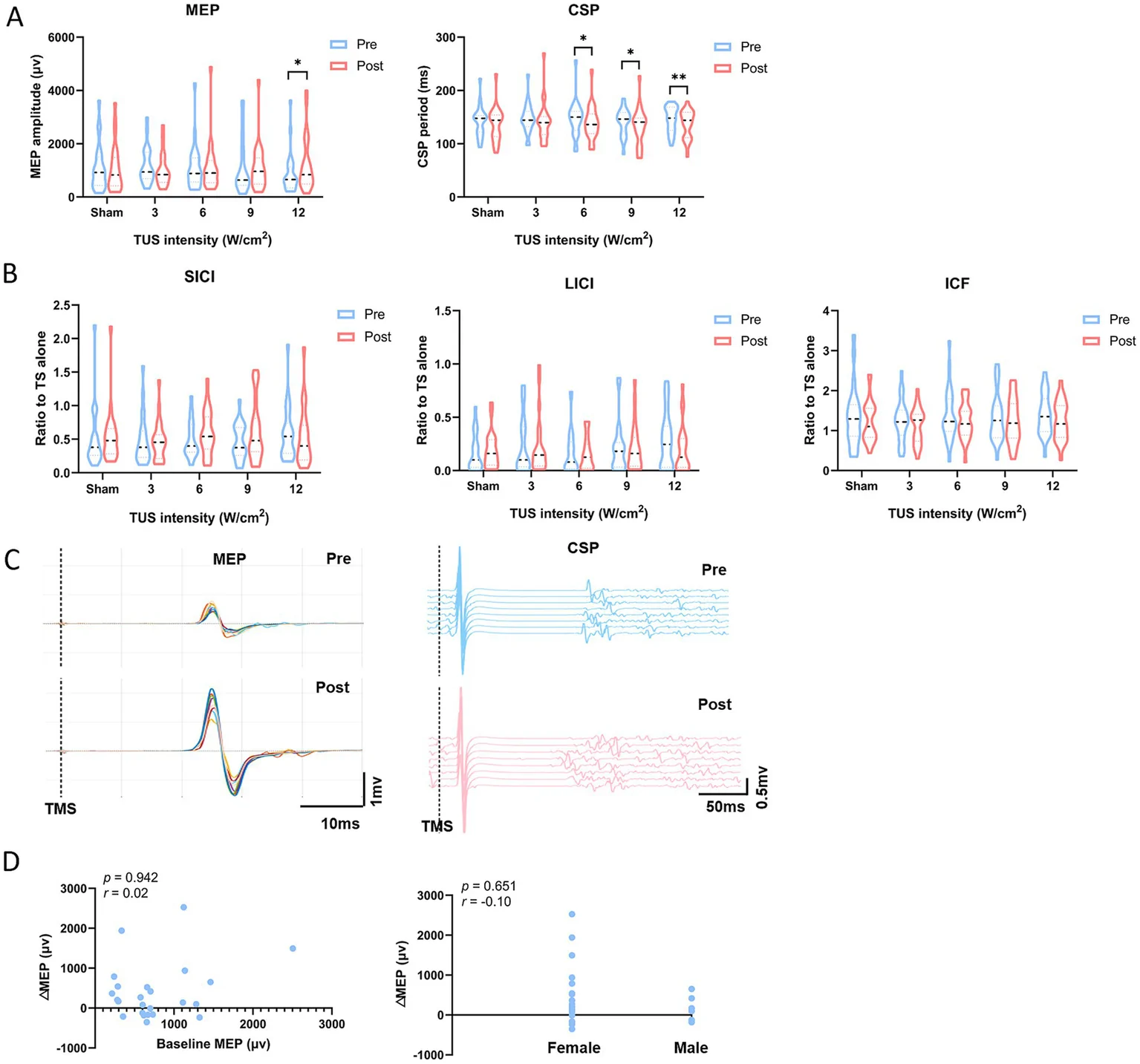
The effects of TUS on motor cortical excitability. **(A)** Mean MEP amplitudes and cSP durations at baseline and post sonication for sham and four active TUS intensity conditions. **(B)** Mean paired pulse TMS measures (SICI, LICI, and ICF) at baseline and post sonication for all five conditions. Values are expressed as a ratio to the MEP amplitude elicited by the test stimulus (TS) alone. **(C)** Representative MEP and cSP waveforms from a single participant before and after TUS at an intensity of 12 W/cm^2^. **(D)** Correlation plots examining the relationship between the TUS induced change in MEP amplitude (△MEP) at 12 W/cm^2^ and either baseline MEP amplitude or gender. Error bars represent the standard error of the mean. Asterisks denote statistical significance: *p < 0.05, **p < 0.01.

To evaluate the effect of TUS on motor cortex excitability, we analyzed several TMS-EMG metrics before and after sonication across five experimental sessions. A one way ANOVA confirmed that baseline MEP amplitudes did not differ significantly across the five sessions prior to intervention [*F* (4, 24) = 0.435, *p* = 0.574], indicating consistent baseline corticospinal excitability. The mean (SD) baseline MEP amplitudes were 1.16 mV (0.66) for 3 W/cm^2^, 1.12 mV (0.85) for 6 W/cm^2^, 1.13 mV (1.03) for 9 W/cm^2^, 0.88 mV (0.77) for 12 W/cm^2^, and 1.08 mV (0.85) for the sham condition. A two way repeated measures ANOVA on MEP amplitudes revealed a significant Time × Condition [*F* (4, 96) = 6.268, *p* = 0.019], while the main effects of Time [*F* (1, 24) = 0.302, *p* = 0.588], and Condition [*F* (4, 96) = 0.086, *p* = 0.987] were not significant. Post-hoc comparisons revealed that sonication at 12 W/cm^2^ induced a significant increase in MEP amplitude from pre-TUS to post-TUS (*p* = 0.036). No significant changes in MEP amplitude were observed in any other active or sham condition (Figure 3A).

For the cSP, baseline durations were consistent across all sessions, with no significant differences observed [*F* (4,24) = 0.435, *p* = 0.574]. The mean (SD) baseline cSP durations were 147.56 ms (27.41), 147.31 ms (32.95), 141.25 ms (24.50), 145.73 ms (23.98), and 141.76 ms (27.85) for the 3, 6, 9, 12 W/cm^2^, and sham conditions, respectively. The rmANOVA for cSP duration yielded a significant main effect of Time [*F* (1, 24) = 23.382, *p* < 0.001], but no significant main effect of Condition [*F* (4, 96) = 1.131, *p* = 0.347] and no Time × Condition interaction [*F* (4, 96) = 0.222, *p* = 0.926]. Post-hoc paired comparisons revealed that TUS significantly shortened the cSP duration at intensities of 6 W/cm^2^ (*p* = 0.039), 9 W/cm^2^ (*p* = 0.036), and 12 W/cm^2^ (*p* = 0.003). No significant changes were observed for the 3 W/cm^2^ or sham conditions (Figure 3A).

Analysis of intracortical circuits via paired pulse TMS showed no effect of sonication. One-way ANOVA confirmed no significant differences in baseline measures of SICI, LICI and ICF across the five experimental conditions. The subsequent rmANOVA showed no significant Time × Condition interactions for ICF [*F* (2.415, 57.97) = 0.438, *p* = 0.685], SICI [*F* (4, 96) = 0.267, *p* = 0.898] or LICI [*F* (1.738, 41.701) = 1.06, *p* = 0.347]. Furthermore, there were no significant main effect of Time or Condition on any of these three measures (all *p* > 0.05), as illustrated in Figure 3B.

### Correlation analysis

To investigate potential factors influencing the observed excitatory effect, we performed correlation analyses. Spearman’s correlation analysis showed no significant relationship (*r* = 0.01565, *p* = 0.9421) between the baseline MEP amplitude and the change in MEP (△MEP) following sonication at an ISPPA of 12 W/cm^2^ (Figure 3D). Similarly, a point-biserial correlation analysis revealed that gender was not correlated with the △MEP at this intensity (*r* = −0.09731, *p* = 0.651) (Figure 3D).

## Discussion

This study investigated the immediate effects of varying TUS intensities on corticospinal and intracortical excitability within the human primary motor cortex (M1). Our primary finding is that TUS delivered at an ISPPA of 12 W/cm^2^ potentiated MEP amplitudes, indicative of enhanced corticospinal excitability. In contrast, lower intensity (3, 6, and 9 W/cm^2^) and a sham condition produced no such effect. Furthermore, TUS produced a significant shortening of cSP at intensities of 6 W/cm^2^ and above, whereas measures of intracortical inhibition and facilitation (SICI, LICI, and ICF) remained unaltered across all conditions. Notably, the observed excitatory effect was not correlated with baseline MEP amplitude or gender. These results underscored the critical role of ISPPA as a key parameter for achieving excitatory neuromodulation in the human M1 and contribute to the needed parameter optimization of TUS parameters for targeted applications.

The selective potentiation of MEP amplitudes exclusively at the 12 W/cm^2^ intensity, without concurrent changes in SICI, ICF or LICI, suggests a threshold-dependent excitatory mechanism that primarily targets corticospinal output pathways rather than modulating the broader intracortical circuitry. This phenomenon is likely attributable to the mechanical effects of acoustic pressure on neuronal membranes, which can influence the gating of mechanosensitive ion channels, and subsequently alter neuronal firing rates, as demonstrated in preclinical models (Lee et al., 2016; Tipsawat et al., 2022; Kim et al., 2014). At lower ISPPA levels, the delivered acoustic energy may be insufficient to surpass the biophysical threshold required to elicit detectable changes in excitability. This interpretation is consistent with previous work; for example, Huang et al. (2025) observed an intensity dependent transition from no effect to excitation in the human motor cortex, although their reported threshold of 4 W/cm^2^ differs from ours. The discrepancy in the excitatory threshold between our findings and theirs may arise from differences in transducer characteristics, sonication protocols, or the precision of neuronavigated targeting. Our findings are, however, in agreement with the general principle demonstrated by Zeng and colleagues (Zeng et al., 2024), who found that a theta burst TUS protocol had no effect at 10 W/cm^2^ but produced significant excitation at 20 W/cm^2^. In line with this intensity dependent effect, other work (Caffaratti et al., 2025) has also emphasized that higher ISPPA levels favor excitatory outcomes. Collectively, these studies and ours affirm that the precise tuning of ISPPA is a viable strategy for achieving desired excitatory neuromodulatory effects.

A key finding of this study is the consistent shortening of the cSP following TUS at 6, 9, and 12 W/cm^2^ intensity. This observation, coupled with the stability of SICI, ICF, or LICI, pointed to a selective regulatory effect of TUS on specific inhibitory neural circuits within M1. The cSP is thought to be primarily mediated by activity at *γ*-aminobutyric acid type B (GABA_B) receptors (Bai et al., 2023a, 2023b), where SICI is a reflection of GABA_A receptor mediated inhibition. Although LICI also involves GABA_B receptors, it is generated by intracortical circuits that are not identical to those contributing to the cSP (Guerra and Bologna, 2022). The finding that 6 and 9 W/cm^2^ shortened the cSP without increasing MEP amplitude may therefore reflect different effective mechanosensitive thresholds across neuronal populations. Resting MEP amplitude depends on the recruitment and synchronization of a sufficiently large population of deep layer corticospinal neurons, whereas the cSP is measured during voluntary contraction and may be more sensitive to modest changes in the timing or efficacy of GABA_B receptor mediated inhibitory control. Accordingly, lower acoustic intensities may be sufficient to modulate specific GABAergic interneurons or inhibitory inputs to corticospinal neurons, but insufficient to produce the broader corticospinal recruitment required for a detectable increase in MEP amplitude. Differences in mechanosensitive ion channel expression, membrane properties and neuronal morphology may contribute to this differential sensitivity. Supporting the general principle of neuron type selective ultrasound responsiveness, Yu et al. (2021) demonstrated that cortical excitatory and inhibitory neurons exhibit intrinsically different responses to TUS parameters. Although that study examined pulse repetition frequency rather than ISPPA, it provides evidence that distinct neuronal populations may have different acoustic response profiles. The isolated modulation of the cSP may therefore indicate preferential engagement of specific GABA_B receptor related inhibitory circuits rather than a generalized alteration of superficial GABA_A receptor mediated networks. This aligns with the view that TUS activates mechanosensitive ion channels via mechanical forces to indirectly modulate GABA_B receptor function (Yoo et al., 2022). In contrast to the findings of Fomenko et al. (2020), who reported no change in cSP with an ISPPA of 9.26 W/cm^2^, our study observed a significant cSP shortening at an ISPPA as low as 6 W/cm^2^. This discrepancy may stem from our optimized TUS pulse parameters (200 Hz PRF, 6% DC) and longer sonication duration (20 min), which may have produced a stronger cumulative effect on the inhibitory circuits contributing to the cSP.

The absence of significant changes in SICI, LICI, and ICF, measures sensitive to GABAergic and glutamatergic intracortical circuits, is also highly informative. This finding indicates that the observed enhancement of excitability does not involve widespread changes in inhibitory or facilitatory intracortical networks. Instead, the excitatory effect is potentially localized to pyramidal neuron or downstream corticospinal pathways. This finding is consistent with previous literature. For instance, a study (Bai et al., 2023a, 2023b) applying intermittent theta burst stimulation (iTBS) to the motor cortex enhanced MEP amplitudes without affecting ICF or SICI. This aligns with reviews by Guerra and Bologna (2022), which posits that low-intensity TUS can specifically modulate corticospinal pathways without concurrently altering these canonical intracortical measures. It is worth noting that while the change in LICI did not reach statistical significance, a slight trend toward reduced inhibition was observable. It remains possible that a larger sample size or higher sonication intensities could reveal significant effects on LICI in future investigations.

The lack of a significant correlation between baseline MEP amplitude and the TUS induced MEP change (ΔMEP) at 12 W/cm^2^ suggests that the excitatory response was not strongly dependent on baseline corticospinal excitability. However, the absence of a significant association between gender and the change in MEP should be interpreted cautiously. Because the sample included 19 females and only 6 males, the analysis had limited statistical power to detect gender related differences. The relatively consistent response may be partly attributable to our use of neuronavigation and standardized stimulation parameters, which minimized inter-subject variability in acoustic energy delivery and targeting precision (Yaakub et al., 2023). Theoretically, one might expect a negative correlation between baseline excitability and response magnitude due to ceiling effects; however, our data suggest that TUS at this intensity may operate through a more direct mechanism, such as the activation of mechanosensitive ion channels, which is less dependent on the pre-existing synaptic state (Yoo et al., 2022). Nevertheless, the unbalanced gender distribution limits the generalizability of these findings, and future studies with larger and more balanced samples are required to determine whether gender influences individual responsiveness to TUS.

Collectively, these findings have significant implications for both neuroscience research and clinical applications. From a basic science perspective, they help refine models of ultrasound neuron interaction by demonstrating an intensity dependent threshold for excitation and a selective modulation of inhibitory circuits. From a clinical standpoint, the identification of specific excitatory parameters opens avenues for developing therapeutic TUS protocols for disorders characterized by hypoexcitable motor cortices, such as in motor recovery after stroke (Huang et al., 2025), PD (Samuel et al., 2023), and DOC (Cain et al., 2022). However, at the molecular level, abnormal alternative RNA splicing is increasingly recognized as an important mechanism in neurodegenerative diseases, suggesting that future studies should explore whether repeated TUS influences activity related molecular pathways associated with neuronal maintenance and recovery (Ran et al., 2026). At the network level, motor recovery after subcortical stroke may involve severity dependent reorganization of interhemispheric pathways, including compensatory interactions between the contralesional dorsal premotor cortex and subcortical networks, which may inform network reorganization for TUS interventions (Fan et al., 2026). Future research should determine the duration of these effects, optimize stimulation strategies for long term treatment, and investigate TUS in neurological disorders to clarify its effects on molecular mechanisms and network function.

## Limitations and future directions

While the present study establishes a clear intensity dependent excitatory effect in healthy young adults, it is important to consider certain limitations. First, our findings are specific to a cohort of healthy young individuals, and the generalizability of the optimal 12 W/cm^2^ intensity to elderly individuals or patient populations remains to be determined. Age related changes in skull properties and cortical plasticity could alter the intensity dependent relationship. Second, our experimental design was tailored to capture the immediate, off-line effects of sonication, and consequently did not assess the potential for inducing longer-term neuroplastic changes. Third, the acoustic intensities reported was quantified in free water and do not account for the inevitable attenuation and phase aberration of the acoustic beam by the skull.

These limitations highlight several critical avenues for future investigation. Longitudinal studies employing multi session protocols are a necessary next step to determine whether these acute excitatory effects can be consolidated into sustained neuroplasticity and translate into tangible clinical benefits. Future work would also be greatly strengthened by incorporating subject specific acoustic simulations based on CT and MRI data to provide a more accurate estimation of the acoustic energy delivered to the target. Furthermore, integrating TUS with functional neuroimaging and behavioral assessments would allow for mapping the broader network level consequences and translate its effect into observable behavioral changes. Ultimately, employing the promising excitatory parameters identified here to effective clinical interventions will require systematic investigation of efficacy and safety within neuropsychiatric disorders populations. Furthermore, psychiatric disorders such as depression also involve systemic metabolic dysfunction through interactions between the brain and body, as well as neurodevelopmental morphological adaptations associated with cell specific gene expression (Lian et al., 2026; Kuang et al., 2023). Therefore, future targeted TUS strategies may need to consider these multisystemic and transcriptomic features when optimizing long term TUS interventions for heterogeneous psychiatric populations.

## Conclusion

In summary, this study demonstrates that low-intensity transcranial ultrasound enhances human motor cortex excitability in a clear intensity-dependent manner. We identify a spatial peak pulse average intensity of 12 W/cm^2^ as a key threshold for inducing immediate potentiation of corticospinal output. Mechanistically, this excitatory effect appears to be highly specific, directly targeting corticospinal pathways while sparing the canonical intracortical circuits responsible for short and long interval inhibition and facilitation. These findings provide foundational evidence for the intensity-specific nature of TUS neuromodulation and advance our ability to develop rational, evidence-based therapeutic protocols. Future work should focus on translating these findings into clinical populations and employing functional neuroimaging to elucidate the broader network-level impact of this targeted stimulation, thereby refining TUS as a precision tool for treating neurological disorders.

## Data Availability

The original contributions presented in the study are included in the article/supplementary material, further inquiries can be directed to the corresponding authors.
